# Global status and trends of exosomes in neurodegenerative diseases from 2014 to 2023: a bibliometric and visual analysis

**DOI:** 10.3389/fnagi.2025.1496252

**Published:** 2025-03-11

**Authors:** Hao Wu, Yao-lei Li, Pan-miao Liu, Jian-jun Yang

**Affiliations:** ^1^Department of Anesthesiology, Pain and Perioperative Medicine, The First Affiliated Hospital of Zhengzhou University, Zhengzhou, China; ^2^National Institutes for Food and Drug Control, Beijing, China

**Keywords:** neurodegenerative diseases, exosomes, Alzheimer’s disease, bibliometric, research frontiers

## Abstract

**Background:**

Neurodegenerative diseases (NDs) are chronic and progressive conditions that significantly impact global public health. Recent years have highlighted exosomes as key mechanisms involved in these diseases. This study aims to visualize and analyze the structure and content of exosomes in NDs based on past research to identify new research ideas and directions. Through bibliometric analysis, we assess the current state of research on exosomes in the field of NDs worldwide over the past decade, highlighting significant findings, major research areas, and emerging trends.

**Methods:**

Publications on exosomes in NDs research were obtained from the Web of Science Core Collection (WOSCC) database. Eligible literature was analyzed using Bibliometric R, VOSviewer, and Citespace.

**Results:**

Between 2014 and 2023, 2,393 publications on exosomes in NDs were included in the analysis. The number of relevant publications has been increasing yearly, with China leading in international collaboration, followed by the United States. And China has the largest number of academic scholars as leading and corresponding authors in all the countries, known as the great research society and community. Notable institutions contributing to these publications include *Nia*, *the University of San Francisco California*, and *Capital Medical University*, which rank highly in both publication volume and citations. *Dimitrios Kapogiannis* is a pivotal figure in the author collaboration network, having produced the highest number of publications (Sato et al., 2011) and amassed 3,921 citations. The journal with the most published articles in this field is *The International Journal of Molecular Sciences*, which has published 131 articles and received 3,347 citations. A recent analysis of keyword clusters indicates that “Exosome-like liposomes,” “Independent mechanisms,” and “Therapeutic potential” are emerging research hotspots.

**Conclusion:**

This is the first bibliometric study to provide a comprehensive summary of the research trends and developments regarding exosomes in NDs studies. Future research in this area may explore the role of mesenchymal stromal cells, microRNAs (miRNAs), and targeted drug delivery systems to further investigate the underlying mechanisms and develop new therapeutics.

## Graphical abstract

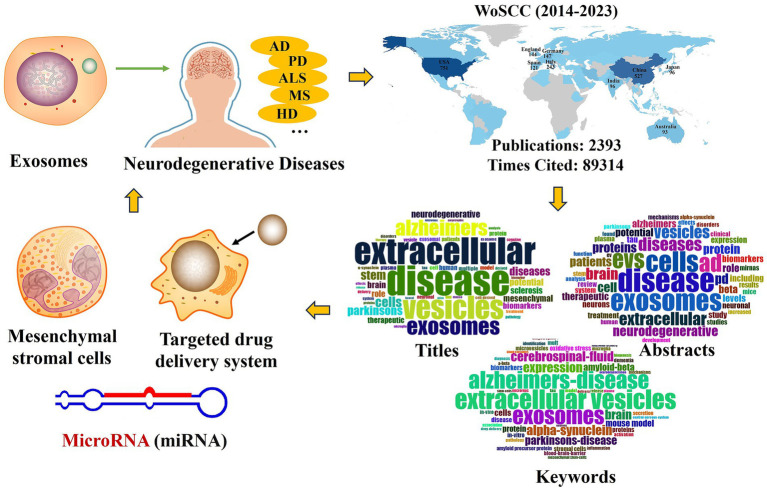

## 1 Introduction

Neurodegenerative diseases (NDs) such as Alzheimer’s disease (AD), Parkinson’s disease (PD), muscle-contracted amyotrophic lateral sclerosis (ALS), multiple sclerosis (MS), and Huntington’s disease (HD) are on the rise each year, becoming a significant global public health issue. There is an urgent need to explore effective prevention and treatment strategies (Dugger and Dickson, 2017; Gale et al., 2018; Melo et al., 2011).

Exosomes are nanoscale extracellular vesicles secreted by cells that facilitate the transfer of specific components from donor cells to recipient cells (Xia et al., 2019; Xia et al., 2022). Exosomes derived from the central nervous system have been shown to cross the blood–brain barrier and enter the systemic circulation (Mathieu et al., 2019; Bellingham et al., 2015). Hey carry unique molecular signatures that reflect changes in cell status and function during disease progression, providing valuable insights into disease pathogenesis (Kim et al., 2014; Yáñez-Mó et al., 2015; Kalra et al., 2012; O’Brien et al., 2013). Currently, the exosomes studied in NDs include neuronal exosomes (Men et al., 2019; Chivet et al., 2014; Goldie et al., 2014; Gong et al., 2016; Sharma et al., 2019), astrocyte exosomes (Pascua-Maestro et al., 2018; Wang et al., 2011; Sollvander et al., 2016), oligodendrocyte exosomes (Kramer-Albers, 2020), and microglial exosomes (Kumar et al., 2017; Huang et al., 2018). Existing studies demonstrated that exosome-labeled proteins are also detected in amyloid *β*-protein (Aβ) plaques in AD brains, suggesting that specific types of exosomes may also trigger the accumulation of Aβ plaques around neurons (Rajendran et al., 2006; Saman et al., 2012). Exogenous neuronal exosomes were continuously injected into the brain of APP transgenic mice, reducing the level of Aβ deposition by enhancing microglia phagocytosis (Yuyama et al., 2014). Additionally, it has been found that *α*-synuclein (*α*-syn), a protein linked to neurodegenerative diseases, is loaded into exosomes released by neurons. These exosomes can transfer *α*-syn between cells and compromise neuronal vitality. Significantly higher levels of *α*-syn have been detected in the blood of PD patients compared to healthy individuals, highlighting the critical role of α-syn-associated exosomes in NDs (Emmanouilidou et al., 2010; Grey et al., 2015).

Bibliometrics is an interdisciplinary science that employs mathematical and statistical methods to quantitatively analyze all forms of knowledge dissemination (Soteriades and Falagas, 2005; Durieux and Gevenois, 2010; Glanville et al., 2010; Tran et al., 2019; Ke et al., 2020). The results derived from bibliometric analysis can evaluate the current state of a research field and help identify emerging research hotspots, thereby providing new avenues for investigation (Zhang et al., 2015; Yang et al., 2018). In recent years, numerous papers have been published regarding exosomes in neurodegenerative diseases (NDs), with studies indicating that exosomes may play a significant role in these conditions. Therefore, this study aims to conduct a bibliometric analysis of publications related to exosomes in NDs from 2014 to 2023. The objectives are to identify the main contributors, assess the current research landscape, and evaluate trends and prospects in this area of this field research.

## 2 Materials and methods

### 2.1 Data source

A literature search was conducted on December 31, 2023, focusing on relevant publications from January 1, 2014, onwards. The search was performed using the Web of Science Core Collection database (WoSCC).^1^ The search strategy was defined as follows: [topic search = (Exosomes OR Extracellular Vesicles) AND (multiple sclerosis OR amyotrophic lateral sclerosis OR Parkinson OR Alzheimer OR Huntington OR Neurodegenerative)]. Publications released before January 1, 2014, or not written in English were excluded from the results. To maintain the integrity of the research in this field, other types of articles were also excluded, such as meeting abstracts, editorial materials, letters, proceedings papers, corrections, early access articles, book chapters, news items, reprints, retracted publications, and retractions. This query yielded a total of 2,393 publications.

### 2.2 Data collection and analysis

All data retrieved from the (WoSCC) were exported using various bibliometric analysis software for research purposes. VOSviewer was employed to analyze partnerships among countries, institutions, and authors of highly cited literature. It also creates a visual map of the network and clusters keywords with high co-occurrence frequency to identify research hotspots and trends. CiteSpace captures keywords with significant citation bursts, analyzes citations for journals and clusters, and visually maps all entries. Citation surges are a crucial indicator for identifying emerging trends in research within this field. Bibliometrix.org was utilized to construct a global distribution network of publications related to exosomes in neurodegenerative diseases (NDs).

## 3 Results

### 3.1 Publication output and temporal trend

**Figure 1:**
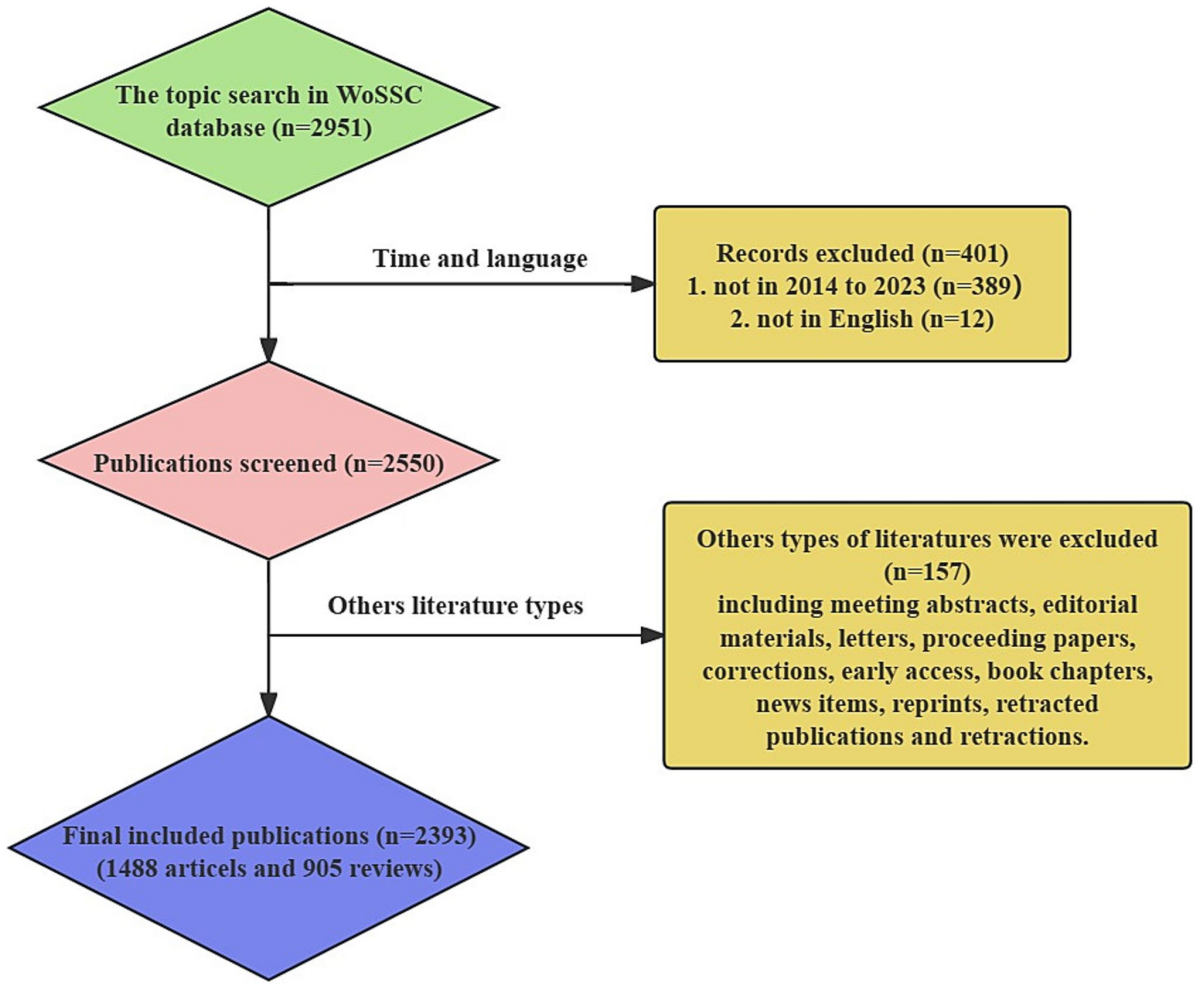
Flowchart of data filtration processing and excluding publications.

**Figure 2:**
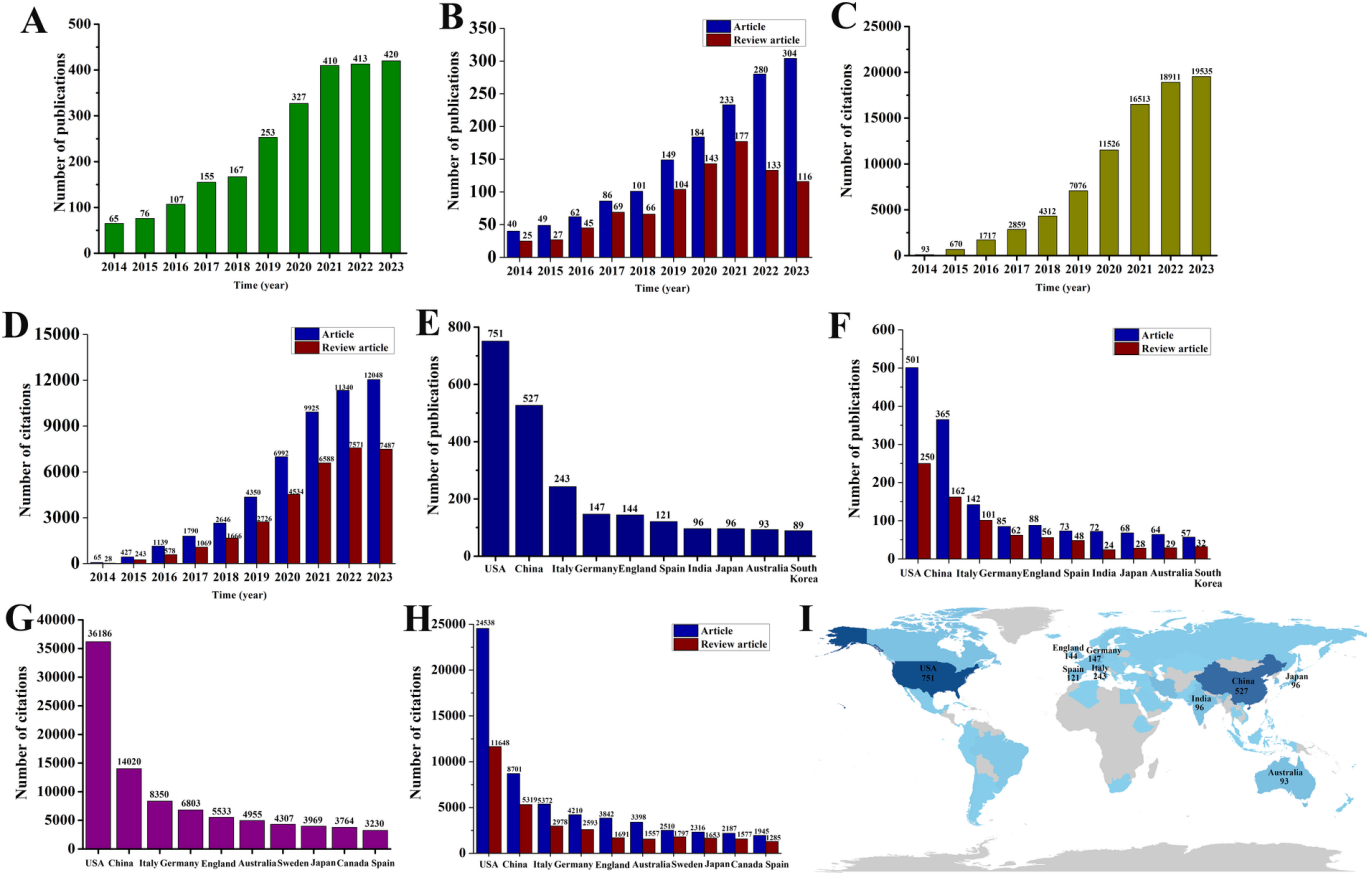
Trends in the number of publications and analysis of country of exosomes in NDs research. **(A,B)** The annual national publication (including articles and review articles) output of the 10 most productive countries. **(C,D)** The 10 most-cited (including articles and review articles) countries. **(E,F)** The annual worldwide publication (including articles and review articles) output. **(G,H)** The annual worldwide citations (including articles and review articles) output. **(I)** Regional distribution.

After conducting a thorough search and assessment, a total of 2,393 publications met the inclusion criteria. This includes 1,488 articles and 905 review articles (Figure 1). Since 2014, the amount of research on exosomes in the field of neurodegenerative diseases (NDs) has steadily increased, peaking in 2023 with 304 articles and 116 review articles (Figures 2A,B). The total number of citations for these publications has also seen a yearly increase, reaching 18,911 in 2023, which includes 12,048 citations for articles and 7,287 for review articles (Figures 2C,D). When analyzing the number of publications and citations by country, the United States leads with 751 publications (501 articles and 250 review articles), followed by China with 365 publications (203 articles and 162 review articles) and Italy with 243 publications (142 articles and 101 review articles) (Figures 2E,F). Additionally, the United States has the highest number of citations, totaling 36,186 (including 24,538 for articles and 11,648 for review articles), while China follows with 14,020 citations (8,701 for articles and 5,319 for review articles). There are notable gaps in the publication and citation counts for other countries (Figures 2G,H). A global visualization map illustrates the depth of study for each country within this research area (Figure 2I).

### 3.2 Academic collaboration

**Figure 3:**
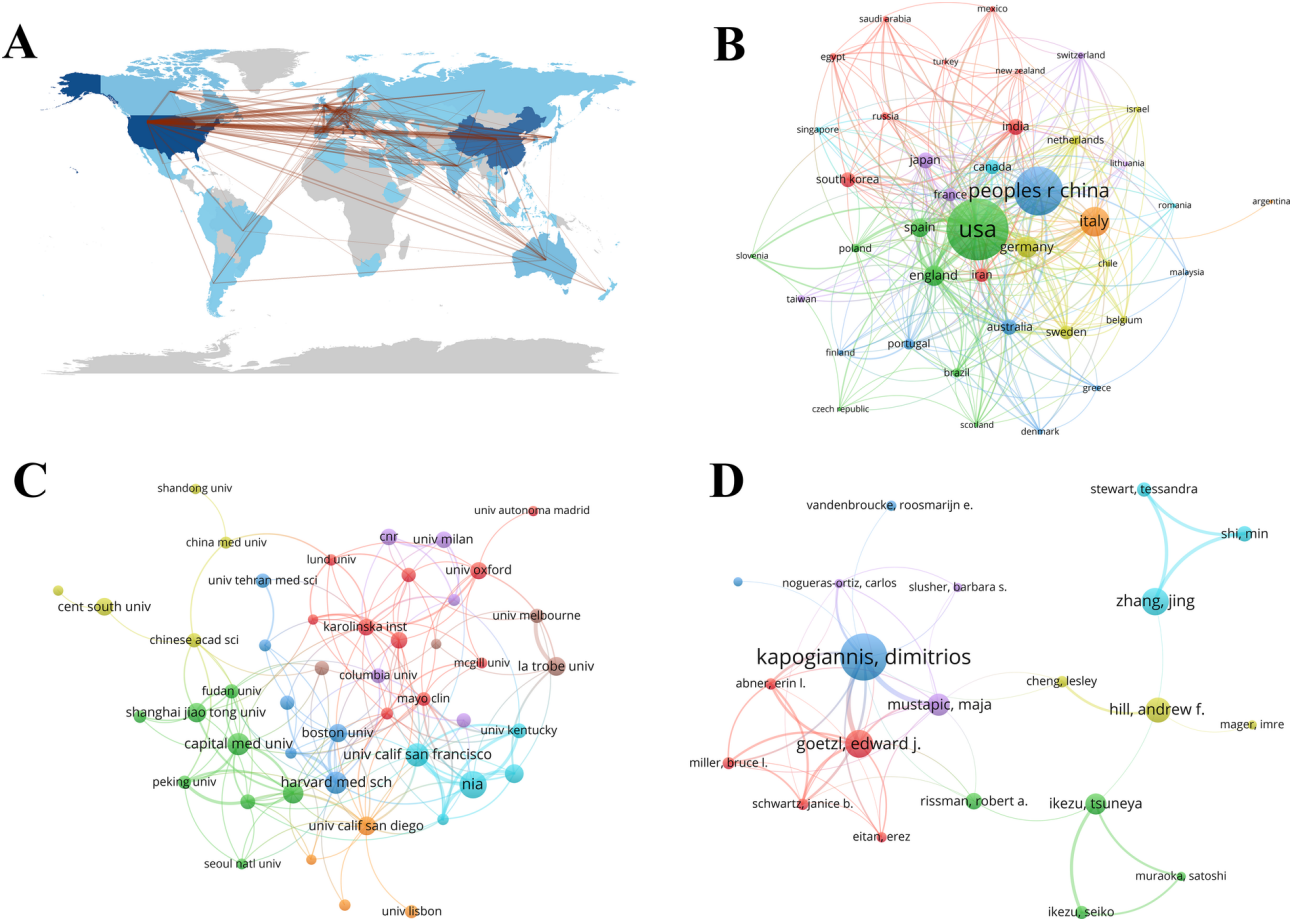
Academic collaboration between countries, institutions, and authors of exosomes in NDs research. **(A,B)** Collaboration of countries and geographical map. **(C)** Collaboration of institutions. **(D)** Collaboration of authors.

Academic collaborations between different countries, institutions, and authors play a crucial role in facilitating knowledge exchange and expanding research on exosomes in neurological disorders (NDs). The geographic map in Figure 3A clearly illustrates the extensive cooperation among various countries, with China and the United States leading international partnerships. According to Figure 3B, China, the USA, and Italy rank as the top three countries engaging in collaborations with others. 47 institutions have published more than 15 papers each, as shown in Figure 3C. Notably, Nia, the University of San Francisco, Capital Medical University, Harvard Medical School, Zhejiang University, and Shanghai Jiao Tong University have each published over 34 papers, significantly contributing to research in this field (Table 1). The top 10 most productive authors in exosome research related to NDs from 2014 to 2023 are listed in Table 2. Kapogiannis and Dimitrios are identified as pivotal figures within the author cooperation network, having published the highest number of papers (Sato et al., 2011) and amassing a total of 3,921 citations (Figure 3D).

**Table 1:** The top 10 productive institutions of exosomes in NDs research from 2014 to 2023.

Rank	Institution	Publication	Article	Review article	Citations
1	Nia	53	38	15	4,239
2	University of California	43	29	14	2,857
3	Capital Medical University	41	27	14	1,097
4	Harvard Medical School	41	30	11	1,670
5	Zhejiang University	37	25	12	1,261
6	Shanghai Jiao Tong University	34	24	10	1,183
7	Johns Hopkins University	33	21	12	2,613
8	La Trobe University	32	21	11	2,157
9	University of California, San Diego	32	20	12	1,677
10	Boston University	31	21	10	2,525

**Table 2:** The top 10 productive authors of exosomes in NDs research from 2014 to 2023.

Rank	Authors	Publication	Article	Review article	Citations	Total link strength
1	kapogiannis, dimitrios	47	34	13	3,921	82
2	goetzl, edward j.	27	19	8	3,013	63
3	zhang, jing	26	19	7	1,487	28
4	hill, andrew f.	24	16	8	1731	10
5	mustapic, maja	21	14	7	1,582	44
6	ikezu, tsuneya	20	13	7	1962	23
7	rissman, robert a.	15	9	6	527	7
8	li, yan	14	10	4	460	4
9	shi, min	14	9	5	1,015	26
10	levy, efrat	13	8	5	489	10

### 3.3 Category and local citation analysis

**Figure 4:**
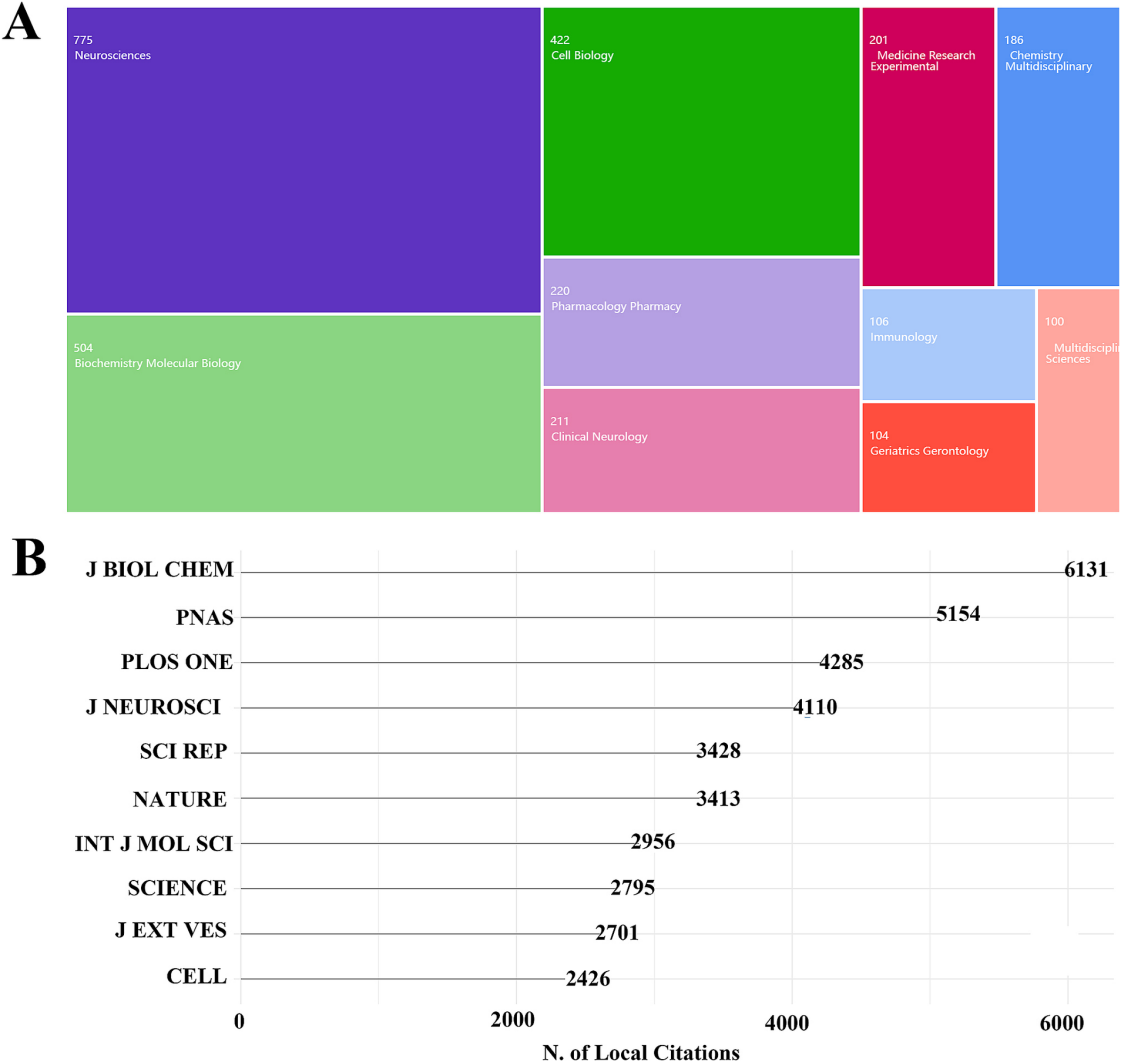
Analysis of the subject category of exosomes in NDs research. **(A)** Distribution of subject categories. **(B)** Local citation analysis.

The topic categories of exosomes in neurodegenerative disease (ND) studies were analyzed across all relevant literature. Figure 4A presents a tree diagram illustrating the top 10 categories in the Web of Science (WOS), along with the number of corresponding articles. The top three categories account for over 70% of the total publications: Neurosciences with 775 papers (32.39%), Biochemistry and Molecular Biology with 504 papers (21.06%), and Cell Biology with 422 papers (17.63%).

An analysis of the 2,393 publications on exosomes in ND research revealed the most productive journals, which are listed in Table 3. After examining the subject categories, Figure 4B displays the top 10 journals based on local citations, with the numbers indicating the citation counts. The results show that the *Journal of Biological Chemistry* has the highest number of local citations, totaling 6,163. The second-ranked journal is *PLoS ONE*, with 5,154 local citations, followed by the *Journal of Neuroscience*, which has 4,110 citations. Renowned international journals such as *Nature*, *Science*, and *Cell* also rank highly in ND research.

**Table 3:** The top 10 productive journals of exosomes in NDs research from 2014 to 2023.

Rank	Journals	Documents	Citations	IF (2023)	Category quartile
1	International journal of molecular sciences	131	3,347	5.6	Q1
2	Cells	67	1,375	6	Q2
3	Frontiers in aging neuroscience	45	1,176	4.8	Q2
4	Frontiers in neuroscience	45	2,557	4.3	Q2
5	Journal of alzheimers disease	45	1,245	4	Q2
6	Frontiers in molecular neuroscience	41	1,177	4.8	Q2
7	Molecular neurobiology	40	1,440	5.1	Q2
8	Scientific reports	34	1,288	4.6	Q2
9	Frontiers in cell and developmental biology	33	672	5.5	Q1
10	Frontiers in immunology	31	1,280	7.3	Q1

### 3.4 Keyword detection and burst analysis

**Figure 5:**
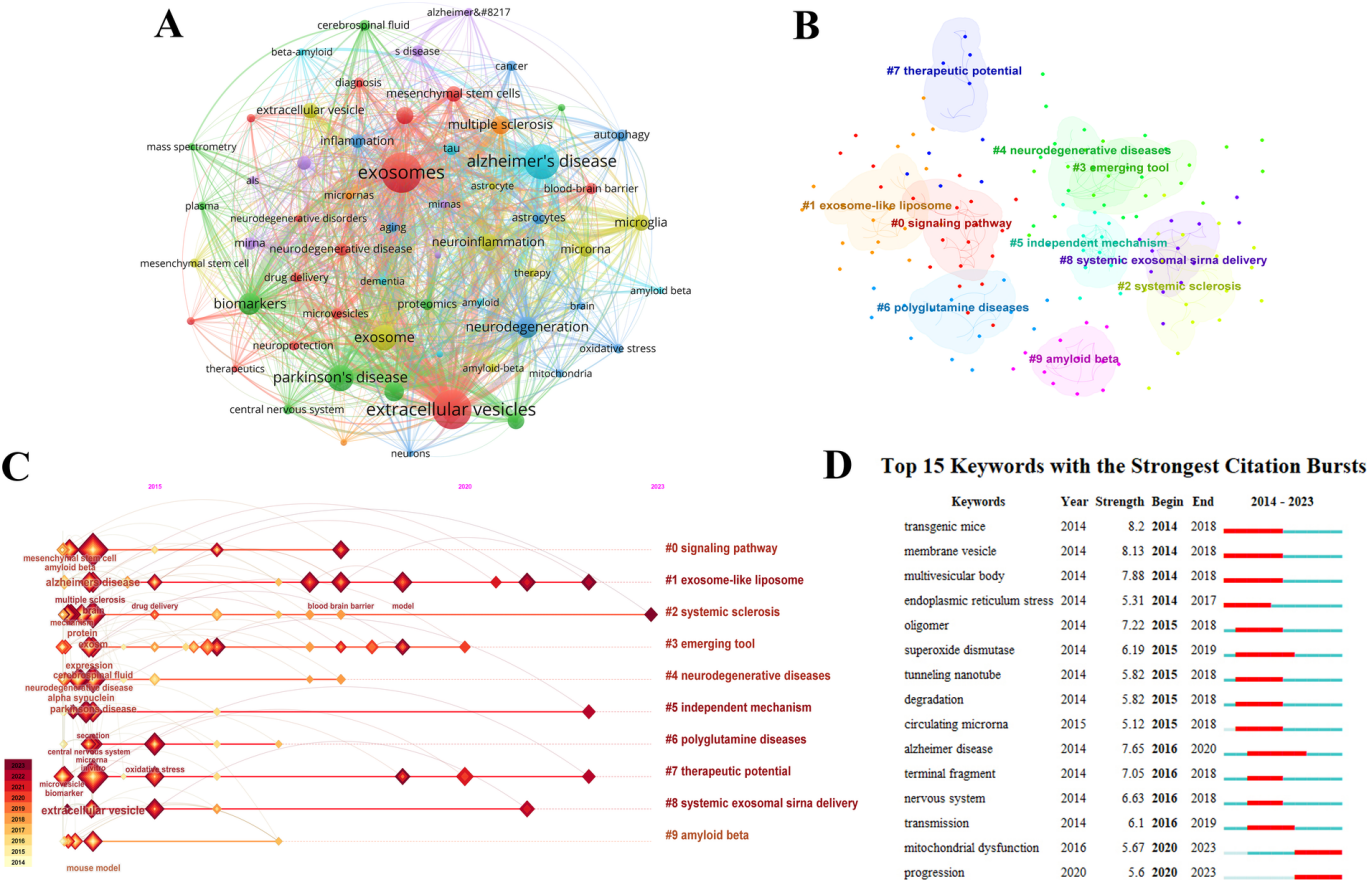
Keyword analysis of exosomes in NDs research. **(A)** Co-occurrence analysis of keywords. **(B)** Keyword clustering. **(C)** Keyword timeline analysis. **(D)** Burst analysis of the top 15 keywords.

Keywords can highlight the key areas and emerging trends in specific fields. A total of 60 keywords each appearing more than 20 times were selected for co-occurrence cluster analysis using VOSviewer. The 10 most frequent keywords identified were: Exosomes (581 occurrences), Extracellular vesicles (545 occurrences), Alzheimer’s disease (442 occurrences), Parkinson’s disease (255 occurrences), Exosome (237 occurrences), Biomarkers (182 occurrences), Neurodegeneration (170 occurrences), Multiple sclerosis (133 occurrences), Neurodegenerative diseases (114 occurrences), and Neuroinflammation (113 occurrences) (Table 4 and Figure 5A). Citespace was utilized for keyword clustering and timeline analysis

**Table 4:** The top 10 keywords of exosomes in NDs research from 2014 to 2023.

Rank	Keyword	Occurrences	Total link strength
1	Exosomes	581	1,279
2	Extracellular vesicles	545	1,198
3	Alzheimer’s disease	442	958
4	Parkinson’s disease	255	591
5	Exosome	237	453
6	Biomarkers	182	471
7	Neurodegeneration	170	440
8	Biomarker	147	367
9	Multiple sclerosis	133	279
10	Neurodegenerative diseases	114	261

The analysis revealed the following top clusters: #0 Signaling pathway, #1 Exosome-like liposome, #2 Systemic sclerosis, #3 Emerging tool, #4 Neurodegenerative diseases, #5 Independent mechanism, #6 Polyglutamine diseases, #7 Therapeutic potential, #8 Systemic exosomal siRNA delivery, and #9 Amyloid beta. A timeline analysis showed that Exosome-like liposomes, Independent mechanisms, and Therapeutic potential have emerged as prominent research hotspots in recent years and are currently significant research directions (Figures 5B,C).

Burst analysis of keywords sheds light on the fastest-growing topics in recent years. As illustrated in Figure 5D, the intensity of the top 15 keywords experiencing an outbreak ranged from 5.6 to 8.2, with durations spanning from 3 to 4 years. Among these, transgenic mice exhibited the highest outbreak intensity, lasting for 5 years.

### 3.5 Co-citation analysis of references

The analysis of 2,393 publications on exosomes in NDs research revealed the 10 most academically influential papers (Table 5). A co-citation relationship occurs when two or more papers are cited together in a single publication. By examining the bursts and clusters of co-cited references, CiteSpace can summarize the research directions among the referenced documents and identify common research themes.

**Table 5:** The top 10 publications in terms of citations related to exosomes in NDs research from 2014 to 2023.

Rank	Title	Year	Type	Autor	Journal	IF	Citations
1	Exosomes	2019	Review	Pegtel, DM	Annual Review of Biochemistry	16.6	1,428
2	Exosomes as drug delivery vehicles for Parkinson’s disease therapy	2015	Review	Haney, MJ	Journal of Controlled Release	10.8	1,264
3	Depletion of microglia and inhibition of exosome synthesis halt tau propagation	2015	Article	Asai, H	Nature Neuroscience	25	1,006
4	Identification of preclinical Alzheimer’s disease by a profile of pathogenic proteins in neurally derived blood exosomes: A case–control study	2015	Article	Fiandaca, MS	Alzheimer’s & Dementia	14	585
5	A new pathway for mitochondrial quality control: mitochondrial-derived vesicles	2014	Article	Sugiura, A	The EMBO Journal	11.4	477
6	Extracellular vesicles round off communication in the nervous system	2016	Review	Budnik, V	Nature Reviews Neuroscience	34.7	476
7	Emerging roles of exosomes in normal and pathological conditions: new insights for diagnosis and therapeutic applications	2015	Article	De Toro, J	Frontiers in Immunology	7.3	461
8	Plasma exosomal α-synuclein is likely CNS-derived and increased in Parkinson’s disease	2014	Article	Shi, M	Acta Neuropathologica	12.7	438
9	The release and trans-synaptic transmission of Tau via exosomes	2017	Article	Wang, Y	Molecular Neurodegeneration	15.1	428
10	Designer exosomes produced by implanted cells intracerebrally deliver therapeutic cargo for Parkinson’s disease treatment	2018	Article	Kojima, R	Nature Communications	16.6	404

**Figure 6:**
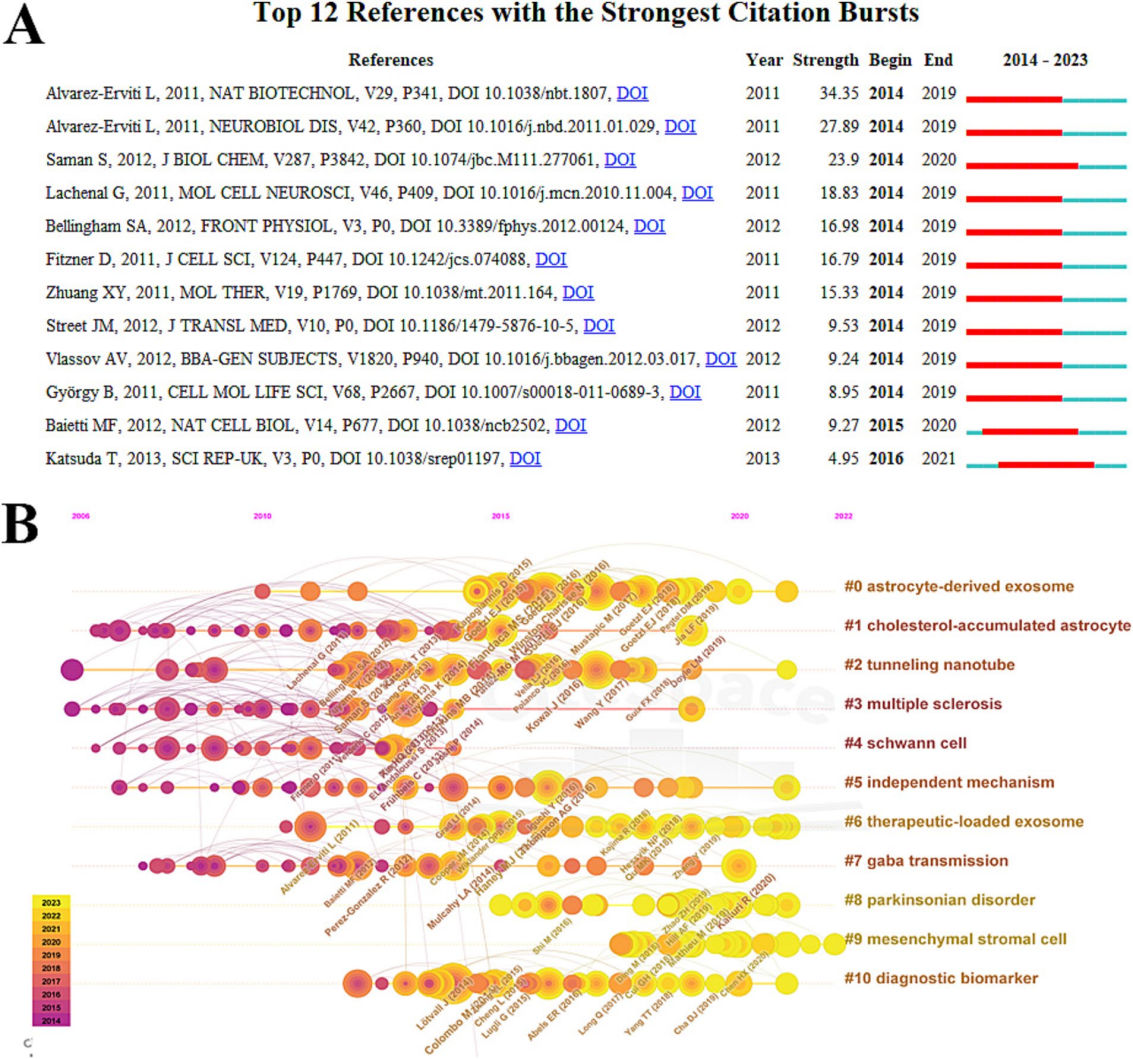
Co-citation analysis of exosomes in NDs research. **(A)** Burst analysis in the top 12 co-cited references. **(B)** Timeline view of co-citation.

Figure 6A displays the top 12 references with the most significant bursts in citation activity. Notably, a 2011 publication in *Nature Biotechnology* by Alvarez-Erviti et al. showed the highest explosive power with an intensity of 34.35 (Alvarez-Erviti et al., 2011b). The same year, another influential paper published in *Neurobiology of Disease* had an intensity of 27.89, underscoring the importance of Alvarez-Erviti et al.’s findings on exosomes in ND research and establishing a solid reference for future analyses (Alvarez-Erviti et al., 2011a). Additionally, a 2012 study by Saman et al. in the *Journal of Biological Chemistry* demonstrated strong explosive power, with an intensity of 23.90 (Saman et al., 2012). Other highly ranked publications also made significant contributions to the advancement of the field (Table 6) (Lachenal et al., 2011; Bellingham et al., 2012; Fitzner et al., 2011; Zhuang et al., 2011; Street et al., 2012; Vlassov et al., 2012; György et al., 2011; Baietti et al., 2012; Katsuda et al., 2013).

**Table 6:** The top 10 co-cited references related to exosomes in NDs research from 2014 to 2023.

Rank	Title	Year	Type	Autor	Journal	IF	Citations
1	Delivery of siRNA to the mouse brain by systemic injection of targeted exosomes	2011	Review	Alvarez-Erviti L	Nature Biotechnology	46.9	3,251
2	Lysosomal dysfunction increases exosome-mediated alpha-synuclein release and transmission	2011	Review	Alvarez-Erviti L	Neurobiology of Disease	6.1	537
3	Exosome-associated tau is secreted in tauopathy models and is selectively phosphorylated in cerebrospinal fluid in early Alzheimer disease	2012	Article	Saman S	Journal of Biological Chemistry	4.8	708
4	Release of exosomes from differentiated neurons and its regulation by synaptic glutamatergic activity	2011	Article	Lachenal G	Molecular and Cellular Neuroscience	3.5	414
5	Exosomes: vehicles for the transfer of toxic proteins associated with neurodegenerative diseases?	2012	Review	Bellingham SA	Frontiers in Physiology	4	292
6	Selective transfer of exosomes from oligodendrocytes to microglia by macropinocytosis	2011	Review	Fitzner D	Journal of Cell Science	4	594
7	Treatment of brain inflammatory diseases by delivering exosome encapsulated anti-inflammatory drugs from the nasal region to the brain	2011	Review	Zhuang X	Molecular Therapy	12.4	1,001
8	Identification and proteomic profiling of exosomes in human cerebrospinal fluid	2012	Article	Street JM	Journal of Translational Medicine	7.4	353
9	Exosomes: current knowledge of their composition, biological functions, and diagnostic and therapeutic potentials	2012	Article	Vlassov AV	Biochimica et Biophysica Acta	3	1,489
10	Membrane vesicles, current state-of-the-art: emerging role of extracellular vesicles	2011	Article	György B	Cellular and Molecular Life Sciences	8	1,549

The analysis of the co-cited timeline view reveals the temporal relationships of hot topics by examining the co-cited references of the included papers. This method highlights research frontiers in the field. The timeline view of the top 10 key clusters is presented, and the results demonstrate significant clustering, indicated by a modularity value (Q) greater than 0.3 and an average contour value exceeding 0.7. In this context, the location of each occurrence corresponds to the time of its first citation, while a larger circle radius signifies a higher number of citations. This analysis suggests that the hot topics in exosome research concerning neurodegenerative diseases (NDs) are evolving. The identified clusters include: Astrocyte-derived exosomes (cluster #0), cholesterol-accumulated astrocytes (cluster #1), tunneling nanotubes (cluster #2), multiple sclerosis (cluster #3), Schwann cells (cluster #4), independent mechanisms (cluster #5), therapeutic loaded exosomes (cluster #6), GABA transmission (cluster #7), Parkinsonian disorders (cluster #8), mesenchymal stromal cells (cluster #9), and diagnostic biomarkers (cluster #10).

### 3.6 Topics evolution and future outlook

**Figure 7:**
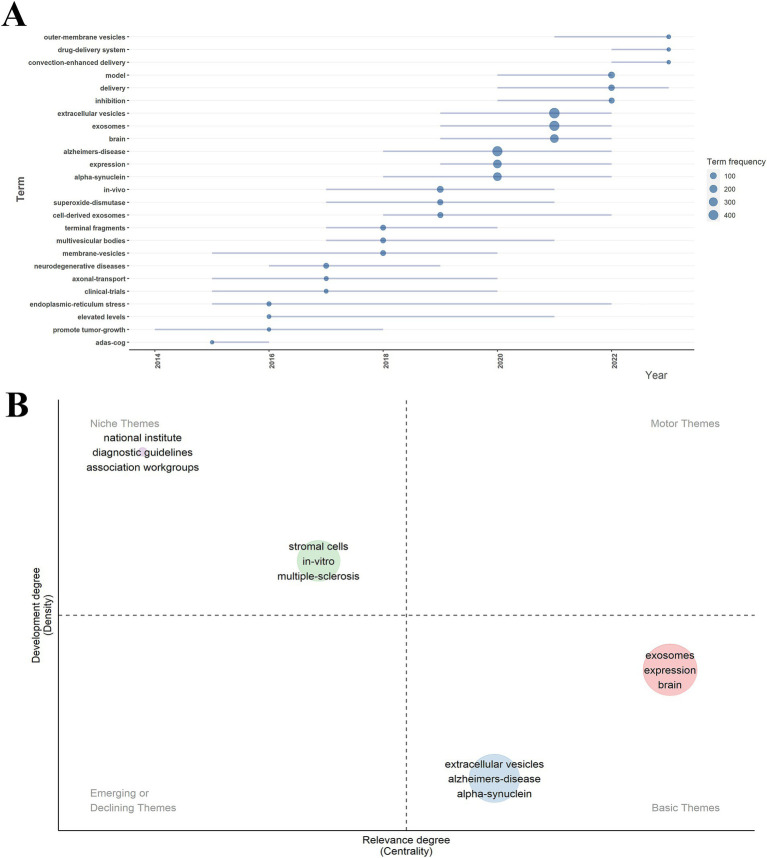
Trend topics map and thematic map of exosomes in NDs research. **(A)** Trend topics map of keywords from 2014 to 2023. **(B)** Thematic map.

This analysis examines trending keyword topics from the last 10 years using R-bibliometrix. As illustrated in Figure 7A, the horizontal axis represents the year of occurrence, while the vertical axis indicates the frequency of keyword occurrences. The size of each node reflects how often a keyword appears, and the length of the horizontal line shows the duration of each keyword’s relevance. From 2013 to 2022, the keyword “extracellular vesicles” had the highest frequency, followed by “exosomes” and “brain.” Notably, “alpha-synuclein” garnered significant attention between 2018 and 2022. The latest trending themes regarding exosomes in neurodegenerative diseases (NDs) include outer-membrane vesicles, drug delivery systems, and convection-enhanced delivery.

Figure 7B presents a thematic map of exosome research in NDs. The relative position of each concept on the map indicates the development stage of the theme: (1) mature and essential themes are located in the upper middle region (e.g., stromal cells, *in vitro* studies, multiple sclerosis); (2) highly developed themes are found in the middle right region (e.g., exosomes, expression, brain); and (3) emerging themes are located in the lower right region (e.g., extracellular vesicles, Alzheimer’s disease, alpha-synuclein).

## 4 Discussion

### 4.1 General information

This bibliometric analysis revealed that the primary categories of exosome research in the field of neurodegenerative diseases (NDs) are Neurosciences, Biochemistry and Molecular Biology, and Cell Biology. Notably, the journal that published the most articles on this topic is the International Journal of Molecular Sciences, with a total of 131 articles and 3,347 citations, making it the top-ranked journal in terms of citations. Another significant journal in this field is Biochemistry and Molecular Biology.

Additionally, our separate analysis of articles and reviews found that, since 2021, while the total number of published articles has been steadily increasing, the number of published reviews has been declining. This trend suggests that current research on exosomes in the context of NDs is focusing more on new technologies and methods rather than on review analyses, indicating rapid development within this field.

In terms of international collaboration in NDs research related to exosomes, there is significant cooperation among different countries, institutions, and authors. China is the top country in cooperation and exchange with other countries and most of the publications cited are authored by Chinese. Academic cooperation between different countries can promote knowledge exchange and broaden the research horizon of exosomes in NDs, which also reflects China’s important position in this field of research. China and the United States have the most international cooperation. China, the United States and Italy are the top three countries that cooperate with other countries. Capital Med univ., Zhejiang univ., and Shanghai Jiao Tong univ. have all published more than 34 papers, making outstanding contributions to the research in this field. China has the largest number of academic scholars as leading and corresponding authors in all the countries, known as the great research society and community.

A standard research theme can be identified by analyzing the burst and clustering of co-cited references. In 2011, Alvarez-Erviti L et al. published two highly influential papers: one in Nature Biotechnology (intensity = 34.35) and the other in Neurobiology of Disease (intensity = 27.89). These papers demonstrate that exosomes, as explored by Alvarez-Erviti L, play a crucial role in the study of neurodegenerative diseases (NDs). Recent studies indicate that exosomes can influence NDs by regulating microRNA. Additionally, keyword cluster 8 suggests similar findings. Stromal cells, which are mentioned in the same co-cited references, rank highly on the topographic maps of exosomes studied in NDs, indicating their significant role in research within this area. As of 2023, the latest trend regarding exosomes in NDs focuses on drug delivery systems, highlighting the potential of exosomes to transport drugs. This aligns with future research directions exploring the use of exosomes for treating NDs. Furthermore, alpha-synuclein is a key cluster identified alongside the keyword trend theme and clustering of cited references, which may suggest a new research focus on exosomes in the context of NDs.

### 4.2 Hotspots and frontiers

Co-occurrence analysis and burst analysis of keywords and co-cited documents are widely used methods for identifying research hotspots and predicting emerging research frontiers. In this study, we visually track the thematic evolution and prospects of exosomes in NDs research from multiple perspectives. Cluster analysis and thematic mapping indicate that stromal cells are a primary focus in this field (Figures 6B, 7B). The trending themes derived from the keywords suggest that siRNA delivery has gained significant attention in recent years (Figures 5B,C). Additionally, targeted drug delivery systems have emerged as the latest area of interest regarding exosomes in ND research, aligning with the current understanding of exosome delivery mechanisms. Overall, future research frontiers are likely to focus on the following areas:

#### 4.2.1 Mesenchymal stromal cells

Mesenchymal stem cells (MSCs) possess a unique ability to differentiate into various cell lineages, including neurons and glial cells. This characteristic positions MSCs as valuable tools in nervous system research (Sato et al., 2011; Kanczler et al., 2019; Kaundal et al., 2018; He et al., 2019; Takov et al., 2020). Recent studies indicate that exosomes secreted by MSCs can play a therapeutic role in neurodegenerative diseases (NDs) through two primary mechanisms: the first is to directly regulate the pathological process of the disease through miRNA carried by MSCS; the second is to load specific RNA, protein, and small molecule drugs into the exosomes, and use the modified exosomes to target the treatment of nervous system diseases (Zheng et al., 2020; Chopra et al., 2019; Armstrong et al., 2017). Research has demonstrated that MSC-derived exosomes can reduce the prevalence of reactive oxygen species in the hippocampal neurons of Alzheimer’s disease (AD) models, thereby protecting neurons from oxidative damage caused by amyloid-beta (Aβ) proteins (de Godoy et al., 2018). In transgenic mouse models of AD, therapy with MSC exosomes has significantly delayed the progression of cognitive impairment and has had a notable protective effect on cognitive function. This suggests that such therapies could play a critical role in slowing the decline in the quality of life for AD patients throughout the course of the disease (Cui et al., 2018). Furthermore, MSC exosomes have been shown to decrease the levels of pro-apoptotic factors while increasing the levels of anti-apoptotic factors. Beyond inhibiting neuronal apoptosis, MSC exosomes also significantly enhance the regeneration of axons in damaged neurons (Ding et al., 2018). The natural bioavailability and biological properties of exosomes make them promising vectors for the treatment of neurodegenerative diseases.

#### 4.2.2 MicroRNAs

MicroRNAs (miRNAs) are a class of small non-coding RNAs, typically consisting of 20 to 24 nucleotides. Numerous studies have demonstrated that exosomes can play a role in the development of NDs by regulating miRNAs (Soares Martins et al., 2021; Su et al., 2022; Lukiw, 2023; Hill, 2019; Cui et al., 2019; Yang et al., 2020). Currently, miRNAs are implicated in various pathophysiological processes associated with NDs, including neuroinflammation, mitochondrial dysfunction, oxidative stress, and the formation of abnormal proteins (Chen et al., 2019; Upadhya et al., 2020; Visconte et al., 2023; Garcia et al., 2022). BACE1 is a critical enzyme in the production of amyloid-beta (Aβ), which is significant in Alzheimer’s disease (AD). Targeting the inhibition of BACE1 to decrease Aβ levels in the brains of AD patients has been a focus of clinical trials for AD treatment. Recent studies have identified several miRNAs, including miR-9, miR-107, miR-29, miR-124, and miR-195, that negatively regulate BACE1 expression (Xie et al., 2017; Nelson and Wang, 2010; Yang et al., 2015; Zhu et al., 2012). Research by Wang et al. found that high levels of miR-124 in an AD model resulted in decreased expression of PTPN1, leading to a reduction in dendritic spine density and impairing the learning and memory capabilities of the mice (Wang et al., 2018). Additionally, recent investigations have confirmed that the therapeutic effects of mesenchymal stem cell (MSC) exosomes on AD are associated with miR-21. When comparing the therapeutic impact of miR-21 exosomes to that of miR-21- MSC exosomes in AD transgenic mice, it was observed that miR-21 + MSC exosomes could effectively regulate the balance between pro-inflammatory and anti-inflammatory factors, as well as promote the growth of synapses and axons. In contrast, miR-21 - MSC exosomes did not exhibit these beneficial effects (Lee et al., 2018). Given that miRNAs are highly expressed in the brain and have become a focal point of research in central nervous system diseases, the ability of exosomes to regulate miRNAs suggests a promising direction for future research. Investigating how exosomes from various sources can therapeutically affect NDs by modulating miRNAs may lead to new treatment avenues.

#### 4.2.3 Targeted drug delivery systems

Exosomes are nanoscale extracellular vesicles that facilitate the transfer of specific components between donor and recipient cells. Recent studies have demonstrated that exosomes produced by the central nervous system can cross the blood–brain barrier and enter systemic circulation (Colombo et al., 2014; György et al., 2011; Desrochers et al., 2016). This unique property has led to the exploration of exosomes as a means of cell-to-cell communication in neurological diseases (NDs), paving the way for the development of exosomal therapies aimed at promoting recovery from these conditions. Exosomes are considered ideal drug delivery vehicles due to their ability to carry a variety of proteins and RNAs that can be targeted to specific cells, along with their good tolerability within the body (de Jong et al., 2019; Alvarez-Erviti et al., 2011b; Soares et al., 2015; Rajendran et al., 2014; Basso and Bonetto, 2016). For instance, Huo et al. encapsulated a small molecule called Slb within macrophage-derived exosomes (Exo-Slb). Upon entering the brain, Exo-Slb selectively bound to Aβ1-42, inhibiting the polymerization of Aβ1-44 and mitigating astrocyte-mediated neuronal damage by modulating the NF-κB pathway. This approach effectively improved cognitive deficits in Alzheimer’s disease (AD) model mice (Huo et al., 2021). Additionally, QI et al. developed a plasma exosome loaded with quercetin (Exo-Que), which successfully crossed the blood–brain barrier through specific active targeting between HSP70 and TLR4. This targeting enhanced the brain migration of the drug, thereby increasing the brain’s targeting capability and the bioavailability of quercetin (Qi et al., 2020). Furthermore, Yang et al. introduced a safe and efficient method for delivering antisense oligonucleotides (ASOs) using exosomes. They utilized exosomes derived from bone marrow-derived mesenchymal stem cells (hbmMSC) to encapsulate Exo-ASO4. Their findings indicated that Exo-ASO4 effectively reached the brain parenchyma of a Parkinson’s disease (PD) model, significantly reducing the expression and aggregation of *α*-synuclein. This resulted in remarkable amelioration of dopaminergic neuron degeneration and improvement in locomotor function in the PD model mice (Yang et al., 2021).

### 4.3 Advantages and shortcomings

This study is the first to use bibliometrics to systematically analyze research on exosomes in neurodegenerative diseases (NDs), providing comprehensive guidance for scholars in this field. In our study, we employed three widely used bibliometric tools simultaneously, which enhances the robustness of our data. Additionally, bibliometric analysis offers a broader perspective on current trends and cutting-edge developments compared to traditional reviews. However, this study has some limitations. First, our data were obtained solely from the Web of Science Core Collection (WoSCC) database, which may have resulted in the omission of relevant studies from other databases. Second, we only analyzed papers published in English, meaning that our findings might not represent the complete picture of research in this area. Lastly, due to the complexity of information technology and collaboration, bibliometric methods cannot fully distinguish the contributions of different authors or institutions.

## 5 Conclusion

In conclusion, the significant increase in the number of publications on exosomes in NDs over the past decade highlights the growing contribution of researchers worldwide in this area. The leading countries in this field are China and the United States. However, there is still a need to enhance cooperation and communication among countries and institutions. Future research directions should focus on understanding the mechanisms of endogenous exosomes in the occurrence and development of NDs, which will deepen our understanding of the pathological processes involved. Additionally, exogenous exosomes present notable advantages over traditional drugs and cell therapies in treating NDs, making the study of their therapeutic strategies promising for treatment applications. Key research hotspots may include mesenchymal stromal cells, microRNAs (miRNAs), and targeted drug delivery systems. This study could serve as a valuable reference for researchers in the field of neurodegenerative disease research.

## Data Availability

The original contributions presented in the study are included in the article/supplementary material, further inquiries can be directed to the corresponding author/s.

## Footnotes

^1^ https://www.webofscience.com/
